# A Co-creation Centre for Accessible Rehabilitation Technology

**DOI:** 10.3389/fresc.2021.820929

**Published:** 2022-01-07

**Authors:** Andy Kerr, Madeleine Ann Grealy, Anja Kuschmann, Rosie Rutherford, Philip Rowe

**Affiliations:** ^1^Biomedical Engineering, University of Strathclyde, Glasgow, United Kingdom; ^2^Psychological Sciences and Health, University of Strathclyde, Glasgow, United Kingdom; ^3^Chest Heart and Stroke Scotland, Glasgow, United Kingdom

**Keywords:** rehabilitation, technology, accessible, stroke, co-creation

## Abstract

**Background:** The prevalence of disabling conditions is increasing globally. Rehabilitation improves function and quality of life across many conditions, particularly when applied intensively. The limited workforce, however, cannot deliver evidence-based intensive rehabilitation. By providing individuals with the tools for self- rehabilitation, technology helps bridge the gap between evidence and practise. Few people, however, can access rehabilitation technology. Barriers such as cost, training, education, portability and poor design stand in the way of equitable access. Our group of engineers and researchers have established a centre dedicated to developing accessible technology through close, frequent engagement with users and industry.

**Methods:** The centre employs a co-creation model, coupling engineering and science with user experience and industrial partnerships to develop accessible technology and associated processes. Due to the complexity and size of the challenge the initial focus is stroke. Recruited through a medical charity, participants, with a wide range of disabilities, use prototype and commercial technology during an 8-week rehabilitation programme with supervision from health professionals. The centre includes de-weighting systems, neurostimulation, virtual reality, treadmills, bespoke rehab games, communication apps, powered exercise equipment and gamified resistance equipment. Standard outcome measures (International Classification of Functioning, Disability and Health) are recorded before, during, immediately after, and 3 months after the intervention and used in combination with an interview to design the initial rehabilitation programme, which is reviewed fortnightly. Qualitative methods (surveys and interviews) are used to capture personal experiences of the programme and individual technology and an advisory group of stroke survivors help interpret outcomes to feed into the technology design process. Ethical approval has been granted for a pilot cohort study with stroke survivors, which is currently underway (01/09/2021–31/12/2021) investigating acceptability and feasibility, due to report findings in 2022.

**Discussion:** Through partnerships, research collaborations and a co-creation model a new centre dedicated to the development of accessible rehabilitation technology has been launched and currently undergoing acceptability and feasibility testing with stroke survivors. The centre, through its close engagement with users and industry, has the potential to transform the way rehabilitation technology is developed and help revolutionise the way rehabilitation is delivered.

## Introduction

Since the industrial revolution improvements in disease prevention (lifestyle, diet, housing, sanitation and education) have had a transformative effect on the health and longevity of most humans. This has been matched by cures for many diseases through surgical and drug interventions. Where disease and injury can't be cured there are well-developed nursing and medical procedures to maintain an individual's quality of life and palliative care to support the dying (1).

The undoubted success of these health strategies has resulted in people living longer, healthier lives (2). The ageing population and improved survival rates from diseases like stroke, however, mean levels of disability have increased (3). Rehabilitation is known to improve the quality of life, and function, of people living with disability (4–6) as well as reducing the burden on acute health services (7). It is estimated that 2.41 billion people live with health conditions known to benefit from rehabilitation (8), however, access to recommended levels of rehabilitation is grossly unequal across the globe and even in high income countries are typically well-below guideline levels (9–11).

Technology has long been suggested as a means to bridge the gap between evidence-based rehabilitation and what can realistically be provided by health services (12). While there is undoubted potential in this approach, technology needs to be developed with access as a core principle (13), otherwise the gross inequities in the provision of rehabilitation will persist. Developing accessible technology; user friendly, self-managed, cost effective and available for domestic homes or local leisure centres should be seen as a priority for industry, developers and funders.

In this paper we detail the methods employed by the first engineering department (in Scotland) dedicated to using a co-creation framework for designing rehabilitation technology and outline the initial feasibility study. The intention of this approach is to produce technology that is useful; meaning both effective for recovery and accessible to all who need it.

## Key Questions for Centre

While the research team have a number of questions related to the specific effects of rehabilitation and optimisation of a technological approach to self-rehabilitation, the primary questions for the centre are initially concerned with acceptability and feasibility of the overall approach, including safety, adverse events, participant acceptability, recruitment, required levels of supervision, participant training and motivation.

## Methods

The guiding principle of the centre is co-creation through frequent and intensive user engagement. To enable this we have established a fully operational rehabilitation clinic equipped with commercially available, and prototype, rehabilitation technology, including de-weighting systems, non-invasive neurostimulation, virtual reality, treadmill, bespoke video games and tablet apps, powered exercise and gamified resistance equipment (see Table 1 for details). The clinic is staffed by a rehabilitation professional who designs and supervises an 8-week programme, based on an initial assessment of needs and agreed goals, delivered, exclusively, through the available technology.

**Table 1:** Details of equipment used and corresponding participant goals.

**Equipment**	**Manufacturer**	**Participant goals**							
		**Walking speed endurance skill**	**Arm/hand function**	**Communication**	**Flexibility**	**Cognition**	**Balance**	**General fitness**	**Muscle strength**
Interactive hand/forearm exerciser	Gripable, UK		√	√		√			√
Mirror box	SAEBO, USA		√						
Sensory TENS	Medfit, Taiwan		√						
VR Treadmill	MotekMedical, Netherlands	√		√		√	√	√	√
Power assisted exercise equipment	Shapemaster, UK	√	√		√			√	√
Resistance exercise equipment Leg press and cable pulley	MotekMedical, Netherlands		√				√		√
Upper limb de-weighting system	Prototype		√						√
VR headset	Occulus Quest, Facebook Technologies, USA with Incisiv software, UK		√	√		√	√		
Peg board	Rolyan, UK		√						√
Tablet Apps Tactus,	Apple Inc. USA with TactusTherapy, Canada			√		√			

A pilot cohort study with stroke survivors (*n* = 7) has been designed to test the feasibility of this approach as well as participant acceptability. A mixed methods approach will be used to answer the key questions, including recruitment rates, retention rates, adverse events, programme compliance, level of assistance/supervision required, change in function and participant opinions on specific pieces of technology as well as the overall programme. Recruitment to the pilot commenced on 01/09/2021 and will end on 18/11/2021.

### Participants

Potential participants are recruited through an open invitation distributed to local support groups organised by our medical charity partner, Chest Heart and Stroke Scotland (CHSS). Individuals subsequently contacting the centre are assessed for eligibility based on the criteria detailed in Table 2. The study has institutional ethical approval (UEC20/08).

**Table 2:** Eligibility criteria.

**Inclusion**	**Exclusion**
Diagnosis of stroke	Absolute contraindications to physical activity^*^
Over 18 years old	Recent (past 6 weeks) deterioration in health
Discharged from NHS rehabilitation services	Significant change in medication
Able to attend a 2 h rehabilitation session at least once a week for up to 8 weeks	Currently unwell or showing signs of Covid-19 (persistent cough and raised temperature)
Able to follow instructions in English and provide verbal and/or written feedback	

* *This was determined by a self-report health questionnaire*.

Once eligibility has been established an initial appointment is made to complete the informed consent process and let participants become familiar with the centre and some of the technology they may use. Participants, along with the research team, are also asked to adhere to the institutional Covid-19 restrictions during this period, including social distancing, mask wearing and carrying out two lateral flow tests per week.

### Intervention

**Figure 1:**
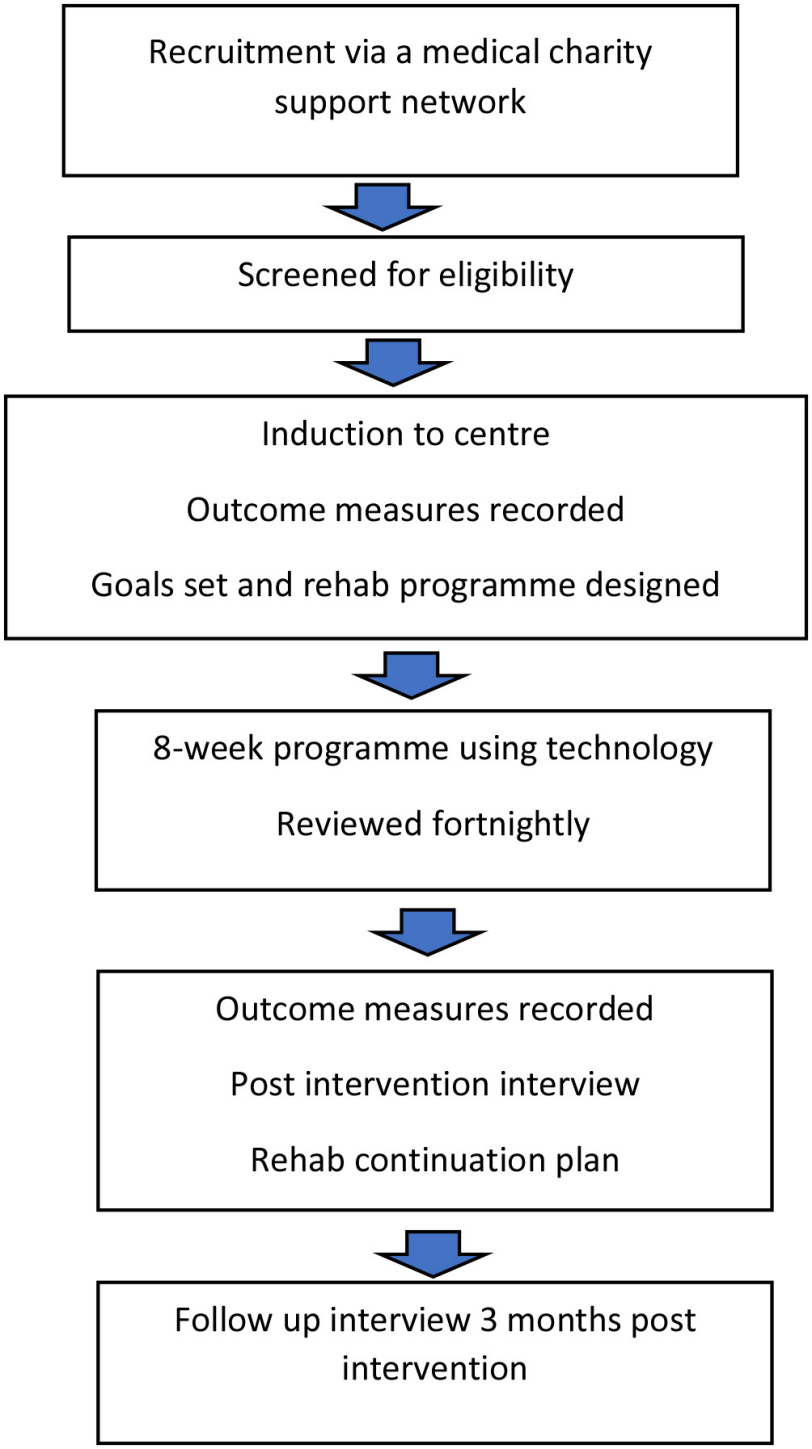
Flow of participants through centre.

The 8-week, technology enabled, rehabilitation programme is structured around the broad principles of intensity, feedback, cognitive engagement and aerobic activity (14) to address the goals identified by the participant and scores recorded from the outcome measures (see Table 3). Rest periods and time to become familiar with the technology are factored into the sessions along with non-invasive priming techniques such as mirror therapy and sensory TENS (29) which participants will be encouraged to use at the start of each session. Figure 1 provides an overview of the flow of participants through the centre.

**Table 3:** Outcome measures used in pilot matched to the ICF framework.

	**References**	**Balance**	**Cognition**	**Communication**	**Function**	**Gait**	**Physical activity**	**Strength**	**Upper limb/hand**	**Quality of life**
Action Research Arm Test (ARAT)	(15)							B	B	
ActivPal monitor	(16)					P	P			
Berg	(17)	B				B				
MOCA	(18)		B							
1 0MWT	(19)	B				B, A				
5 × STS	(20)	B			A			B		
Rivermead Mobility Index	(21)	B			A					
Carer COAST	(22)			B						P
Aphasia Impact Questionnaire 21 (AIQ-21)	(23)			B						P
Functional ambulatory Category	(24)				A					
Stroke Impact Scale (25)	(26)									P
Gait parameters	(27)					B				
Grip strength	(28)							B	B	

*B, Body functions/structure; A, Activity; P, Participation*.

### Outcome Measures

Outcome measures were selected based on their reported acceptability (validity and reliability) in the research literature and common place use in the local health service. Details are provided in Table 3, they represent the body structure (B), activity (A) and participation (P) levels of the ICF (25).

A structured rehabilitation programme will then be developed, co-operatively, by the rehabilitation professional and participant including agreed goals, frequency of attendance, duration and details of individual pieces of equipment to be used, see below for example.

### Example Programme

Total work time 80 min, with rest, as appropriate, in between activities.

### Goals

Improve general fitness, standing balance, lower limb strength, walking (symmetry and speed) and range of upper limb movement.

### Structure

**Figure 2:**
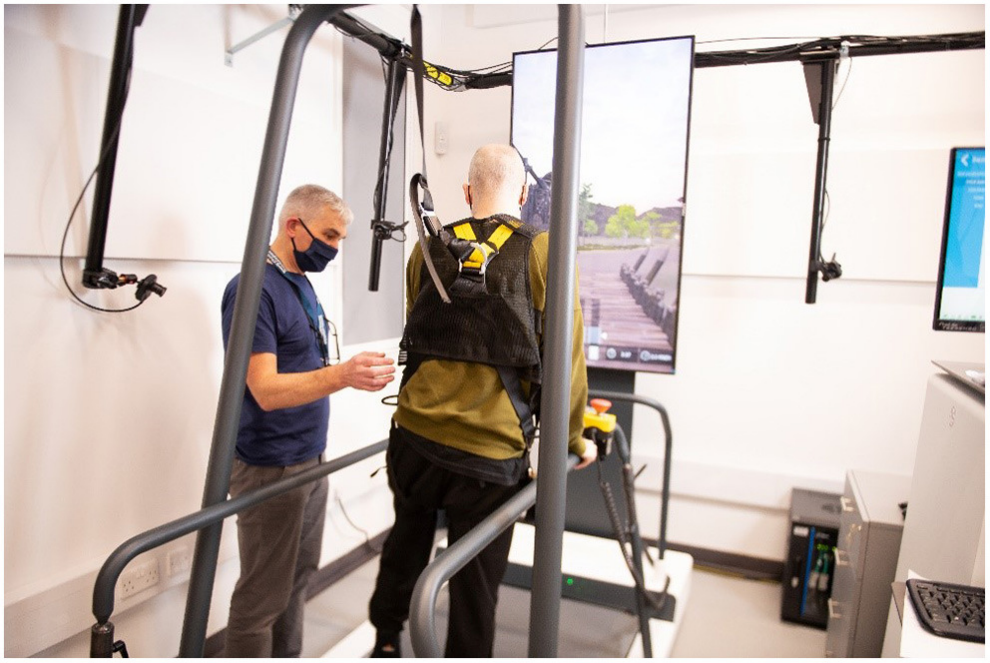
Participant training with an interactive treadmill under supervision.

1. Mirror therapy for hand (20 min)
  Hand/wrist exercises following suggested guideline.
2. Powered exercise equipment
  a. Seated cross trainer, 10 min
  b. Recumbent cycling 5 min.
3. Speech therapy app, picture and word matching, 10 min
4. Treadmill (25 min), (see Figure 2) for illustration
  a. Balance games to encourage increased loading on weaker side, 5 min
  b. Walking in VR environment at comfortable walking speed (0.44 m/s) + 10%, 5 min
  c. Balance games to encourage increased loading on weaker side, 5 min
  d. Walking game that requires frequent adjustments to position, 5 min.
  e. Walking in VR environment at comfortable walking speed with 5 × 20 s periods of fast walking (comfortable walking speed + 20%).
5. Functional squat 10 min—targeting symmetrical loading. 10 Kg load.

### Programme Completion and Exit Interview

Following completion of the 8-week programme the outcome measures are repeated. A plan to continue a modified version of the programme will be discussed. Finally, an independent researcher will arrange a time (within the following 2 weeks) for a one to one interview by telephone or free to download teleconferencing software. The interview will be open with the following questions given as a guideline;

(1) How did you find the treadmill/bike/balance trainer/arm trainer/communication app?

Was there anything you found particularly difficult to manage?

Was there anything you found particularly easy to manage?

(2) Technology based feedback on rehabilitation progress

For example:

Did you find the progress information provided by the equipment useful?

Were you able to access it easily enough?

Did you need help understanding it?

(3) Effectiveness

For example:

Did you achieve your overall goal?

Do you think there has been any change in your communication/mobility/balance/confidence/overall quality of life? Can you give a specific example to describe how this aspect has changed?

Was there any particular piece of equipment that you think helped more?

(4) Need for supervision

For example:

How important do you think having someone present was?

Do you think you need a trained person present?

Was there any equipment you feel you could manage without any help?

(5) Potential for home use

For example:

Would you consider using any of the equipment at home or at your local sports centre?

Would you anticipate any difficulties using this equipment at home or your local sports centre?

What support do you think you might need/like to use this equipment at home?

(6) Future plans

For example:

Now that you have completed the rehabilitation programme will you continue any activities/rehabilitation exercises?

Do you have any plans to continue with rehabilitation?

(7) Ideas for improvement

For example:

Now that you have experienced rehabilitation using machines and equipment do you have any suggestions for improvement?

Thinking about the specific equipment you used do you think we could improve it?

Do you have any ideas of your own that might help people recover from stroke?

How do you think we could improve the overall experience for you?

### Data Analysis

The primary objective for the pilot study is to assess feasibility and acceptability. Data on compliance (sessions completed as percentage of planned), recruitment (people consenting as percentage of those volunteering) and rates of adverse events will be reported with descriptive statistics. Acceptability will be explored from a thematic analysis of the interview transcripts.

In addition any change in the outcome measures (before and after the intervention) will be described with 95% confidence intervals (if data are normally distributed) or interquartile range (if not).

## Discussion

In this paper we have outlined the rationale for a co-creation centre in rehabilitation technology, provided some of the operational detail and outlined the methods for our pilot study designed to test feasibility and acceptability of the overall approach before we plan future research.

Our pilot study is currently underway with seven stroke survivors participating. Once all participants have completed the programme a 6-week review period will be used to collate findings and implement any changes through discussion with our user group of stroke survivors. Once this has been completed, the project will move into a new phase with plans to recruit greater number of participants to our technology enabled rehabilitation programme.

Based on outcomes from the pilot we expect to make changes to operational processes such as recruitment, outcome measures, induction and even the way the individual programmes are structured, however the very positive views expressed by participants to the media, so far (see quotes below for the first two participants), have encouraged us to continue planning future projects, including, but not limited to:

- New devices to improve hand function for more severely affected individuals
- The use of machine learning to tailor individual rehab programmes on a continuous basis
- Recruitment of sub-acute stroke participants.

### Participant Quotes

#1 “I love the sessions, even though they go so quickly. I think the long-term plan is that this unit will act like a drop-in gym for those who need it, which would be fantastic. And if something like this could be available across the country, it would be even better. It would be a shame for people to miss out” (30).

#2 “It was taking me 40 seconds to walk up down the gym and now I can do it in 12 seconds. That's just in the space of a couple of weeks,” (31).

### Potential to Revolutionise Rehabilitation

Despite mounting evidence on the positive effect of rehabilitation, recovery from stroke and other disabling conditions continues to be both disappointing and highly variable (14, 32) leading many researchers to explore the factors that could enable a more personalised, self-managed approach (33), with technology a key enabler (34). If this model of rehabilitation could be widely delivered, removing issues of access, not only would it improve equity but may also realise higher levels of recovery by delivering levels of rehabilitation indicated by research (35).

The optimal “dose” of rehabilitation remains unknown (35), a more technological approach may unlock greater potential for recovery but we, as a community of researchers and engineers, need to understand how this approach can work, equitably, in the real world and continually refine the necessary technology through co-creation with the users.

## Data Availability Statement

The original contributions presented in the study are included in the article/supplementary material, further inquiries can be directed to the corresponding author/s.

## Ethics Statement

The studies involving human participants were reviewed and approved by University of Strathclyde Ethics Committee. The patients/participants provided their written informed consent to participate in this study. Written informed consent was obtained from the individuals for the publication of any potentially identifiable images or data included in this article.

## Author Contributions

All authors listed have made a substantial, direct, and intellectual contribution to the work and approved it for publication.

## Funding

The centre was part funded by the Sir Jules Thorn Charitable Trust.

## Conflict of Interest

The authors declare that the research was conducted in the absence of any commercial or financial relationships that could be construed as a potential conflict of interest.

## Publisher's Note

All claims expressed in this article are solely those of the authors and do not necessarily represent those of their affiliated organizations, or those of the publisher, the editors and the reviewers. Any product that may be evaluated in this article, or claim that may be made by its manufacturer, is not guaranteed or endorsed by the publisher.

## References

[1] Stucki G Bickenbach J Gutenbrunner C Melvin J. Rehabilitation: the health strategy of the 21st century. J Rehabil Med. (2018) 50:309–16. 10.2340/16501977-2200 28140419

[2] WHO. World Health Statistics 2021: Monitoring Health for the SDGs, Sustainable Development Goals. Geneva: WHO (2021).

[3] Chatterji S Byles J Cutler D. Health, functioning, and disability in older adults-present status and future implications (vol 385, pg 563, 2015). Lancet. (2015) 385:508. 10.1016/S0140-6736(14)61462-8 25468158 PMC4882096

[4] Moreau NG Bodkin AW Bjornson K Hobbs A Soileau M Lahasky K. Effectiveness of rehabilitation interventions to improve gait speed in children with cerebral palsy: systematic review and meta-analysis. Phys Ther. (2016) 96:1938–54. 10.2522/ptj.20150401 27313240 PMC5131187

[5] Nguyen C Lefevre-Colau MM Poiraudeau S Rannou F. Rehabilitation (exercise and strength training) and osteoarthritis: a critical narrative review. Ann Phys Rehabil Med. (2016) 59:190–5. 10.1016/j.rehab.2016.02.010 27155923

[6] Platz T. Evidence-based guidelines and clinical pathways in stroke rehabilitation-an international perspective. Front Neurol. (2019) 10:200. 10.3389/fneur.2019.00200 30930832 PMC6423914

[7] Stucki G Stier-Jarmer M Grill E Melvin J. Rationale and principles of early rehabilitation care after an acute injury or illness. Disabil Rehabil. (2005) 27:353–9. 10.1080/09638280400014105 16040536

[8] Cieza A Causey K Kamenov K Hanson SW Chatterji S Vos T. Global estimates of the need for rehabilitation based on the Global Burden of Disease study 2019: a systematic analysis for the Global Burden of Disease Study 2019. Lancet. (2021) 396:2006–17. 10.1016/S0140-6736(20)32340-0 33275908 PMC7811204

[9] Gimigliano F Negrini S. The World Health Organization “Rehabilitation 2030: a call for action”. Eur J Phys Rehabil Med. (2017) 53:155–68. 10.23736/S1973-9087.17.04746-3 28382807

[10] Kerr A Dawson J Robertson C Rowe P Quinn TJ. Sit to stand activity during stroke rehabilitation. Top Stroke Rehabil. (2017) 24:562–6. 10.1080/10749357.2017.1374687 28920550

[11] Bernhardt J Urimubenshi G Gandhi DBC Eng JJ. Stroke rehabilitation in low-income and middle-income countries: a call to action. Lancet. (2020) 396:1452–62. 10.1016/S0140-6736(20)31313-1 33129396

[12] Loureiro RC Harwin WS Nagai K Johnson M. Advances in upper limb stroke rehabilitation: a technology push. Med Biol Eng Comput. (2011) 49:1103–18. 10.1007/s11517-011-0797-0 21773806

[13] Kerr A Smith M Reid L Baillie L. Adoption of stroke rehabilitation technologies by the user community: qualitative study. JMIR Rehabil Assist Technol. (2018) 5:e15. 10.2196/rehab.9219 30120086 PMC6119213

[14] Langhorne P Coupar F Pollock A. Motor recovery after stroke: a systematic review. Lancet Neurol. (2009) 8:741–754. 10.1016/S1474-4422(09)70150-4 19608100

[15] Chen HF Lin KC Wu CY Chen CL. Rasch validation and predictive validity of the action research arm test in patients receiving stroke rehabilitation. Arch Phys Med Rehabil. (2012) 93:1039–45. 10.1016/j.apmr.2011.11.033 22420887

[16] Klenk J Buchele G Lindemann U Kaufmann S Peter R Laszlo R. Concurrent validity of activPAL and activPAL3 accelerometers in older adults. J Aging Phys Act. (2016) 24:444–50. 10.1123/japa.2015-0178 26751290

[17] Blum L Korner-Bitensky N. Usefulness of the berg balance scale in stroke rehabilitation: a systematic review. Phys Ther. (2008) 88:559–66. 10.2522/ptj.20070205 18292215

[18] Wu CY Hung SJ Lin KC Chen KH Chen P Tsay PK. Responsiveness, minimal clinically important difference, and validity of the MoCA in stroke rehabilitation. Occup Ther Int. (2019) 1–7. 10.1155/2019/2517658 31097928 PMC6487084

[19] Persson CU Hansson PO Sunnerhagen KS. Clinical tests performed in acute stroke identify the risk of falling during the first year: postural stroke study in Gothenburg (Postgot). J Rehabil Med. (2011) 43:348–53. 10.2340/16501977-0677 21267528

[20] Mong YQ Teo TW Ng SS. 5-repetition sit-to-stand test in subjects with chronic stroke: reliability and validity. Arch Phys Med Rehabil. (2010) 91:407–13. 10.1016/j.apmr.2009.10.030 20298832

[21] Hsieh CL Hsueh IP Mao HF. Validity and responsiveness of the rivermead mobility index in stroke patients. Scand J Rehabil Med. (2000) 32:140–2. 10.1080/003655000750045497 11028799

[22] Long A Hesketh A Bowen A. Communication outcome after stroke: a new measure of the carer's perspective. Clin Rehabil. (2009) 23:846–56. 10.1177/0269215509336055 19482891

[23] Swinburn K Best W Beeke S Cruice M Smith L Willis EP. A concise patient reported outcome measure for people with aphasia: the aphasia impact questionnaire 21. Aphasiology. (2019) 33:1035–60. 10.1080/02687038.2018.1517406

[24] Mehrholz J Wagner K Rutte K Meissner D Pohl M. Predictive validity and responsiveness of the Functional Ambulation Category in hemiparetic patients after stroke. Arch Phys Med Rehabil. (2007) 88:1314–9. 10.1016/j.apmr.2007.06.764 17908575

[25] Stucki G Cieza A Ewert T Kostanjsek N Chatterji S Ustun TB. Application of the International Classification of Functioning, Disability and Health (ICF) in clinical practice. Disabil Rehabil. (2002) 24:281–2. 10.1080/09638280110105222 12004974

[26] Edwards B O'Connell B. Internal consistency and validity of the stroke impact scale 2.0 (SIS 2.0) and SIS-16 in an Australian sample. Qual Life Res. (2003) 12:1127–35. 10.1023/A:1026109920478 14651430

[27] Balasubramanian CK Neptune RR Kautz SA. Variability in spatiotemporal step characteristics and its relationship to walking performance post-stroke. Gait Post. (2009) 29:408–14. 10.1016/j.gaitpost.2008.10.061 19056272 PMC2675553

[28] Bertrand AM Fournier K Brasey MGW Kaiser ML Frischknecht R Diserens K. Reliability of maximal grip strength measurements and grip strength recovery following a stroke. J Hand Ther. (2015) 28:356–63. 10.1016/j.jht.2015.04.004 26206167

[29] Harmsen WJ Bussmann JBJ Selles RW Hurkmans HLP Ribbers GM. A mirror therapy-based action observation protocol to improve motor learning after stroke. Neurorehabil Neural Repair. (2015) 29:509–16. 10.1177/1545968314558598 25416737

[30] Fotheringham. 'Revolution in Stroke Care' - New Partnership Boost for Glasgow Charity Up for Share of £20k. Glasgow: Glasgow Evening Times.

[31] Cameron C (Director) (2021). New Research Improving Rehabilitation for Stroke Survivors.

[32] Gelaw AY Janakiraman B Gebremeskel BF Ravichandran H. Effectiveness of home-based rehabilitation in improving physical function of persons with stroke and other physical disability: a systematic review of randomized controlled trials. J Stroke Cerebrovasc Dis. (2020) 29:1–8. 10.1016/j.jstrokecerebrovasdis.2020.104800 32278534

[33] Wouters EFM Wouters B Augustin IML Houben-Wilke S Vanfleteren L Franssen FME. Personalised pulmonary rehabilitation in COPD. Eur Respir Rev. (2018) 27:147. 10.1183/16000617.0125-2017 29592864 PMC9488569

[34] Harris NR Sthapit D. Towards a personalised rehabilitation system for post stroke treatment. In: 2016 IEEE Sensors Applications Symposium (Sas 2016) Proceedings. (2016). p. 217–21.

[35] Lang CE Lohse KR Birkenmeier RL. Dose and timing in neurorehabilitation: prescribing motor therapy after stroke. Curr Opin Neurol. (2015) 28:549–55. 10.1097/WCO.0000000000000256 26402404 PMC4643742

